# Noise-induced cochlear synaptopathy in C57BL/6 N mice as a function of trauma strength: ribbons are more vulnerable than postsynapses

**DOI:** 10.3389/fncel.2024.1465216

**Published:** 2024-10-01

**Authors:** Kerstin Blum, Pauline Schepsky, Philip Derleder, Philipp Schätzle, Fahmi Nasri, Philipp Fischer, Jutta Engel, Simone Kurt

**Affiliations:** ^1^Center for Integrative Physiology and Molecular Medicine (CIPMM), School of Medicine, Department of Biophysics, Saarland University, Homburg, Germany; ^2^Center for Gender-specific Biology and Medicine (CGBM), School of Medicine, Saarland University, Homburg, Germany

**Keywords:** noise trauma, cochlear synaptopathy, hidden hearing loss, hair cell, ribbon, postsynapse, auditory nerve, ABR

## Abstract

Noise-induced cochlear synaptopathy is characterized by irreversible loss of synapses between inner hair cells (IHCs) and spiral ganglion neurons (SGNs) despite normal hearing thresholds. We analyzed hearing performance and cochlear structure in C57BL/6 N mice exposed to 100, 106, or 112 dB SPL broadband noise (8–16 kHz) for 2 h. Auditory brainstem responses (ABRs) were assessed before, directly after, and up to 28 days post-trauma. Finally, the number, size, and pairing of IHC presynaptic (CtBP2-positive) ribbons and postsynaptic AMPA receptor scaffold (Homer1-positive) clusters were analyzed along the cochlea. Four weeks after the 100 dB SPL trauma, a permanent threshold shift (PTS) was observed at 45 kHz, which after the higher traumata extended toward middle to low frequencies. Loss in ABR wave I amplitudes scaled with trauma strength indicating loss of functional IHC synaptic connections. Latencies of wave I mostly increased with trauma strength. No trauma-related OHC loss was found. The number of synaptic pairs was reduced in the midbasal and basal cochlear region in all trauma conditions, with ribbon loss amounting up to 46% of control. Ribbons surviving the trauma were paired, whereas 4–6 unpaired postsynapses/IHC were found in the medial, midbasal, and basal regions irrespective of trauma strength, contrasting findings in CBA/CaJ mice. Our data confirm the susceptibility of ribbon synapses and ABR wave I amplitudes to a noise trauma of 100 dB SPL or larger. Notably, peripheral dendrites bearing IHC postsynapses were less vulnerable than presynaptic ribbons in C57BL/6 N mice.

## Introduction

In the adult mammalian cochlea, sound-induced displacement of hair bundles causes depolarization of IHCs and opening of Ca_v_1.3 Ca^2+^ channels (Platzer et al., 2000; Brandt et al., 2003). These channels cluster at 15–30 active zones at the basolateral synaptic pole, which are composed of a single presynaptic ribbon innervated by an afferent dendrite of one SGN (Fuchs et al., 2003; Khimich et al., 2005). Sound-evoked release of glutamate activates glutamate receptors at the afferent boutons and may trigger action potentials in the individual nerve fiber. Research in rodent and primate models has shown that ribbon synapses of IHCs can degenerate after exposure to moderate noise (octave-band noise, 100 dB SPL, 2 h) in the high-frequency region accompanied by a temporary threshold shift (TTS) but without a permanent elevation of hearing thresholds (PTS), which was termed noise-induced hidden hearing loss (NIHHL) (Kujawa and Liberman, 2006, 2009; Hickox et al., 2017). This phenomenon probably also exists in humans (Schaette and McAlpine, 2011; Hickox et al., 2017; Wu et al., 2021).

A functional assay for NIHHL in rodents is the reduction of ABR wave I amplitude, indicating fewer functional nerve fibers contributing to the ABR signal (Kujawa and Liberman, 2009). On the ultrastructural level, an immediate and irreversible reduction of presynaptic ribbons was found in CBA/CaJ mice, which led to the term “cochlear synaptopathy” (Kujawa and Liberman, 2009; Liberman et al., 2015; Liberman and Kujawa, 2017). Similar synaptic damage has been observed in age-related hearing loss in mice (Kujawa and Liberman, 2015; Liberman and Kujawa, 2017; Jeng et al., 2020a).

Whereas ribbon loss in CBA/CaJ mice was irreversible (Kujawa and Liberman, 2009), recent studies revealed partial regeneration of ribbons in C57BL/6 J mice (Shi et al., 2015; Kaur et al., 2019; Manickam et al., 2023) and full regeneration in guinea pigs (Shi et al., 2013; Song et al., 2016; Hickman et al., 2020, 2021). Repair of ribbon synapses requires the action of resident macrophages in the cochlea (Kaur et al., 2019; Manickam et al., 2023).

Noise causes elevated influx of Ca^2+^ through Ca_v_1.3 channels IHCs, which couple IHC membrane potential changes to exocytosis of glutamate (Frank et al., 2009). Excess Ca^2+^ may be toxic to ribbons and cause their degeneration (Kim et al., 2019). Postsynaptically, glutamate excitotoxicity is the accepted reason for swelling, possible detachment, and degeneration of afferent dendrites (Puel et al., 1994; Kujawa and Liberman, 2009; Wang and Green, 2011; Kim et al., 2019).

The ~18 afferent fibers contacting one IHC differ in anatomical and physiological properties and show either high (HSR), medium (MSR), or low spontaneous firing rates (LSRs) (Liberman, 1982). According to molecular differences, HSR fibers correspond to type Ia, MSR fibers to Ib, and LSR fibers to Ic fibers [nomenclature according to Shrestha et al. (2018), with a proportion of Ia: Ib: Ic of approximately 46%: 28%: 26% (Petitpré et al., 2018; Shrestha et al., 2018; Sun et al., 2018)]. HSR fibers are activated at the hearing threshold, LSR fibers at loud tones, and MSR fibers in between; for review, see Moser et al. (2023). It has been suggested that LSR fibers are particularly sensitive to hidden hearing loss and noise-induced cochlear synaptopathy (Furman et al., 2013; Song et al., 2016). Cochlear synaptopathy is also observed after a PTS trauma (Hickox et al., 2017; Valero et al., 2017).

The mouse strain used in this study, C57BL/6 N, has been widely used for the generation of knockout mice by ‘The International Knockout Mouse Consortium’ (Mianné et al., 2016). Our aim was to analyze the effects of the classical NIHHL trauma (TTS only; 8–16 kHz, 2 h, 100 dB SPL) on hearing and cochlear synaptopathy-related symptoms in C57BL/6 N mice and how the outcome was altered by traumata of 106 dB SPL or ml 112 dB SPL, which both cause permanent hearing loss.

## Materials and methods

### Animals

C57BL/6 N mice were purchased from Charles River, Sulzfeld, Germany, and bred in the animal facility of the Centre for Integrative Physiology and Molecular Medicine Homburg, with regular back-crossing to prevent the generation of an in-house substrain. The mice were housed in individually ventilated cages in the temperature-controlled facility with free access to food and water and a 12-h dark/light cycle. Mice of either sex were used for the experiments. The animal care, use, and experimental protocols followed the national and institutional guidelines and were reviewed and approved by the Animal Welfare Commissioner and the Regional Board of Animal Experimentation of Saarland. All experiments were performed in accordance with the European Communities Council Directive (86/609/EEC).

### Auditory brainstem response (ABR) measurements

Mice were anesthetized by injecting a mixture of Fentanyl (0.05 mg/kg body weight, Fentanyl Hameln® - 50 μg/ml, Hameln Pharma Plus GmbH, Hameln, Germany), Midazolam (5 mg/kg body weight, Midazolam Hameln® - 5 mg/ml, Hameln Pharma Plus GmbH, Hameln, Germany), and Medetomidine (0.5 mg/kg body weight, Domitor® - 1 mg/ml, Orion Corporation, Espoo, Finland) intraperitoneally (i.p.) (Deichelbohrer et al., 2017; Julien-Schraermeyer et al., 2020). Surgical tolerance tested with a negative toe pinch reflex was obtained after 15 min for approximately 75 min. If necessary, one-third of the initial dose of anesthetics was injected i.p. to achieve sufficient anesthetic depth. The antidote dosage was a mixture of Naloxone (1.2 mg/kg body weight, Naloxone Inresa® 0.4 mg, Inresa Arzneimittel GmbH, Freiburg, Germany), Flumazenil (0.5 mg/kg body weight, Flumazenil Inresa® – 0.5 mg i.v., Inresa Arzneimittel GmbH, Freiburg, Germany) and Atipamezole (2.5 mg/kg body weight, Antisedan® – 5 mg/ml, Orion Corporation, Espoo, Finland) (Deichelbohrer et al., 2017; Julien-Schraermeyer et al., 2020). The antidote was applied subcutaneously immediately after the end of the ABR measurement. Because of frequent anesthesia, animals were monitored for severity and distress daily for the first 10 days of the experiment according to a score sheet. The score sheet recorded the general condition, wellbeing, spontaneous activity, clinical findings, and body weight. There was a tendency of some weight loss on the day after trauma, which however was not significant. No further deviations were observed. After the final ABR recording, some of the mice were euthanized by cervical dislocation under anesthesia (see above) and used for immunohistochemistry.

ABRs to free field clicks (100 μs) and pure tones (3 ms, 1 ms ramp) were recorded with subdermal silver wire electrodes (diameter: 0.25 mm, purity: 99.99%, Good Fellow, Hamburg, Germany) at the ear (positive), the vertex (negative), and the back of the animal (reference) using the Audiology Lab setup plus software (Otoconsult, Frankfurt, Germany). After amplification by a factor of 10^5^, the signals were averaged for 256 repetitions at each sound pressure level presented (click: 0–80 dB SPL, pure tones: 0–100 dB SPL in steps of 5 dB).

### Noise trauma

Anesthetized animals aged 7–9 weeks were placed in a custom-made sound-proof booth directly underneath the center of a speaker (Stage Line PA Horn Tweeter MHD-220 N/RD, MONACOR INTERNATIONAL GmbH & Co. KG, Bremen) positioned 12 cm above the animal’s head. Body temperature was maintained with a temperature-controlled heating pad (Otoconsult, Frankfurt, Germany). The speaker’s output was measured with a condenser microphone (Brüel & Kjær 4135; Brüel & Kjær, Bremen, Germany) placed at the position of the animal’s head and below the speaker. The microphone output was read in dB SPL with a measuring amplifier (Brüel & Kjær 2636; Brüel & Kjær). The speaker’s frequency spectrum was checked with a spectrum analyzer (Ono Sokki Multi-purpose FFT Analyzer CF-5220; Ono Sokki Technology, Yokohama, Japan). In the 8–16 kHz frequency range of presented noise levels (100, 106, or 112 dB SPL, root-mean-square [SPLrms]), the frequency response was flat (± 2 dB), and no distortion products could be detected. Noise exposure was performed binaurally with octave-band noise (8–16 kHz) at either 100, 106, or 112 dB SPL for 2 h. Noise exposure was interrupted every 30 min for maximally 2 min to check the toe pinch reflex, breathing rate, and to apply additional anesthesia (one third of the initial dose) if required.

### Hearing thresholds and analysis of ABR waveforms

**Figure 1:**
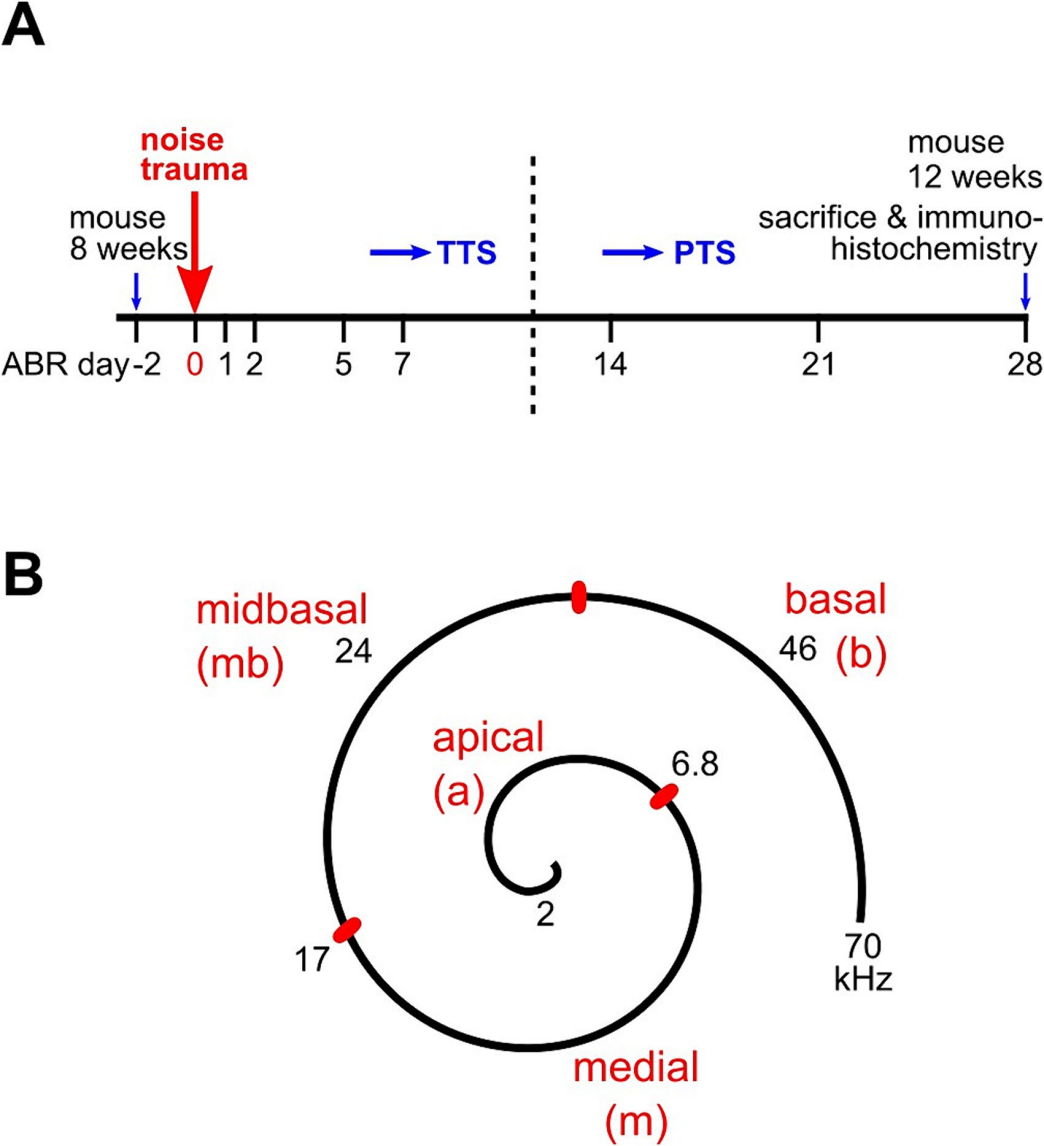
Experimental design of hearing measurements, trauma application, and cochlear assignment. **(A)** ABR thresholds were initially recorded in mice aged 7–8 weeks at day-2 for each animal. At day 0, the animal received a 2 h band noise trauma, 8–16 kHz, of either 100 dB, 106 dB, or 112 dB SPL. Days of further ABR measurements are indicated. ABRs of the no trauma control group were recorded on days-2 and 28 only. After a final ABR recording on day 28, animals were euthanized and 4 of them were processed for immunohistochemistry (see Methods). **(B)** Sketch with the assignment of auditory frequencies to regions of the cochlear spiral in the mouse. Adapted from Müller et al. (2005) and Engel et al. (2006).

Click ABRs and frequency-dependent (f-ABRs) were recorded 2 days before trauma (day-2), directly after the trauma (day 0), and on days 1, 2, 3, 5, 7, 14, 21, and 28 after trauma (Figure 1A). Hearing thresholds were determined by the lowest sound pressure that produced visually distinct evoked potentials by assessing the traces from above threshold to near threshold for each animal and ear. If for a given ear a threshold of the audiogram could not yet be identified at 100 dB SPL, the auditory threshold was defined as 105 dB SPL. For days-2, 0, and 28 after trauma, amplitudes and latencies of ABR wave I were analyzed. The peak-to-peak amplitude of ABR wave I was defined as *I* = *I_n_* - *I_p_*, with *I_n_* being the amplitude of the negative peak at time = *t_n_* and *I_p_* the amplitude of the following positive peak at *t_p_*. Wave I amplitudes as a function of the stimulus level (growth functions) were plotted for all ears. The latency of wave I was defined as *t_n_* with respect to the time of stimulus onset, *t* = 0.

### Immunohistochemistry

Mice were euthanized by cervical dislocation under anesthesia with isoflurane. If not stated otherwise, chemicals were purchased from Sigma-Aldrich (St. Louis, MO, USA). Immunolabeling was performed on whole-mounts of the organ of Corti of 12-week-old mice. Cochlea were dissected from the temporal bone in ice-cold PBS (Gibco, Carlsbad, CA, USA). The scalae of each cochlea were injected with ice-cold Zamboni’s fixative (Morphisto, Offenbach, Germany), followed by immersion in the fixative for 20 min on ice. After replacing the fixative with PBS, the cochlear spiral was carefully dissected into two parts, one containing the apical and medial region covering approximately 45% length of the basilar membrane, and one covering the midbasal and basal part (Figure 1B).

Specimens were permeabilized and blocked with PBS containing 3% BSA and 0.5% Triton-X 100 and incubated in reaction buffer (3% BSA, 0.2% Triton-X in PBS) containing the respective primary antibodies at 4°C overnight. Specimens were labeled with antibodies against CtPB2/Ribeye (mouse monoclonal, BD Transduction Laboratories, Franklin Lakes, NJ, USA; 1:500) and Homer1 (rabbit polyclonal, Synaptic Systems, Germany; 1:1000). Primary antibodies were labeled with Alexa 488-anti mouse secondary antibodies (goat or donkey polyclonal, Fisher Scientific, Carlsbad, CA, USA; 1:500) or Cy3-anti rabbit (goat polyclonal, Jackson Immuno Research Laboratories, Ely, UK; 1:1500) at room temperature for 70 min. Specimens were stained with DAPI at 1:333 in PBS, washed, carefully placed under microscope control, and embedded with VECTASHIELD® H1000 (Vector Laboratories, Newark, CA, USA).

For all immunolabeling experiments, both ears of four animals were analyzed. Specimens were immunolabeled in two or more independent experiments for each condition (control, 100 dB SPL, 106 dB SPL, and 112 dB SPL trauma group). Fluorescence images were acquired using a confocal laser scanning microscope LSM710 with the acquisition software ZEN 2012 SP1, Version 8.1.10.484 (both Carl Zeiss Microscopy GmbH, Jena, Germany). Overview images of 607 μm × 607 μm (1,024 pixel × 1,024 pixel) were obtained using a 20x objective (ZEISS Plan-Apochromat, 0.8 NA), whereas *z*-stacks of optical slices with 70 nm × 70 nm pixel size and 0.32 μm slice thickness were obtained using a 63x oil objective (ZEISS Plan-Apochromat), 1.4 NA, with a pinhole of 1 airy unit. For the quantification of the number and size of ribbons and Homer1 spots, images of 948 × 546 pixels (64.26 μm × 37.01 μm) covering the basolateral poles of usually 8 IHCs on average were acquired at equal laser and gain settings. Images of at least 3 stacks of each part of the organ of Corti were acquired, and the origin of any 8-IHC stack within that part and with regard to its original cochlear tonotopic location was noted by using the overview image of the slide.

### Image processing

Images were processed with Fiji (Schindelin et al., 2012). After calculating the maximum intensity projections (MIPs) of z-stacks, the channel of interest was background-subtracted. If IHCs were positioned very steeply in the organ of Corti, which led to overlay and optical fusion of synapses, these z-stacks were excluded. A thresholded binary image was created (0 below, 1 above threshold) with thresholds of 6–16% of the maximum intensity of the green (CtBP2) and of 5–16% of the red (Homer1) color channel, depending on the individual conditions due to effects of the bony lamina spiralis or of supporting cells on the fluorescence signals, especially in the high-frequency regions of the cochlea. After discarding clusters smaller than 0.05 μm^2^, the number and area of clusters were analyzed by Fiji’s particle count routine. After blinding the data files, the unpaired ribbons and unpaired Homer1 clusters were counted manually. The numbers of pre-and postsynapses were normalized by the number of IHCs in the frame, respectively.

Auditory frequency ranges were assigned to pieces of the organ of Corti according to the murine frequency map (Müller et al., 2005) as follows: 2–6.8 kHz, apical; > 6.8–17 kHz, medial; > 17–32 kHz, midbasal; > 32 kHz, basal (Figure 1B).

### Statistical analysis

Data for ABR thresholds were averaged for each animal; they are presented as mean ± standard deviation (SD) for *n* animals per experimental group. For click and frequency ABRs, differences of the means were tested for statistical significance by the Kruskal–Wallis test (non-parametric) with *α* = 0.05 and Bonferroni test (post-hoc test) using Statistica 13.0 (StatSoft, Hamburg, Germany).

Immunohistochemical data were analyzed using SPSS Statistics Vs. 25.0.0.1 (International Business Machines Corp., Armonk, NY, U.S.A.). Quantitative immunohistochemical data are presented as mean ± SD or as box-and-whisker-plots for small regions of IHCs (usually 8 IHCs per z-stack) of the apical, medial, midbasal, and basal cochlear part, respectively, with images obtained from independent experiments and 4 mice of each trauma group. The homogeneity of variances was tested with the Brown–Forsythe test. In case of equal variances, one-way ANOVA was used; otherwise, the Kruskal–Wallis test. For each cochlear region, data were tested pairwise for the control and a trauma group, respectively, as well as between trauma groups with the Bonferroni post-hoc test. Box-and-whisker plots use boxes to represent the 25th–75th percentiles, horizontal bar to represent the median (the mean is sometimes indicated by a square), and whiskers to represent the 10th–90th percentiles. For better comprehension, the numbers of ribbons, postsynapses, unpaired ribbons or unpaired postsynapses were normalized by the number of IHCs in the particular region, respectively.

## Results

### Noise trauma of increasing strength increases the permanent threshold shift in the high-frequency range and, in addition, causes PTS in middle and lower frequencies

**Figure 2:**
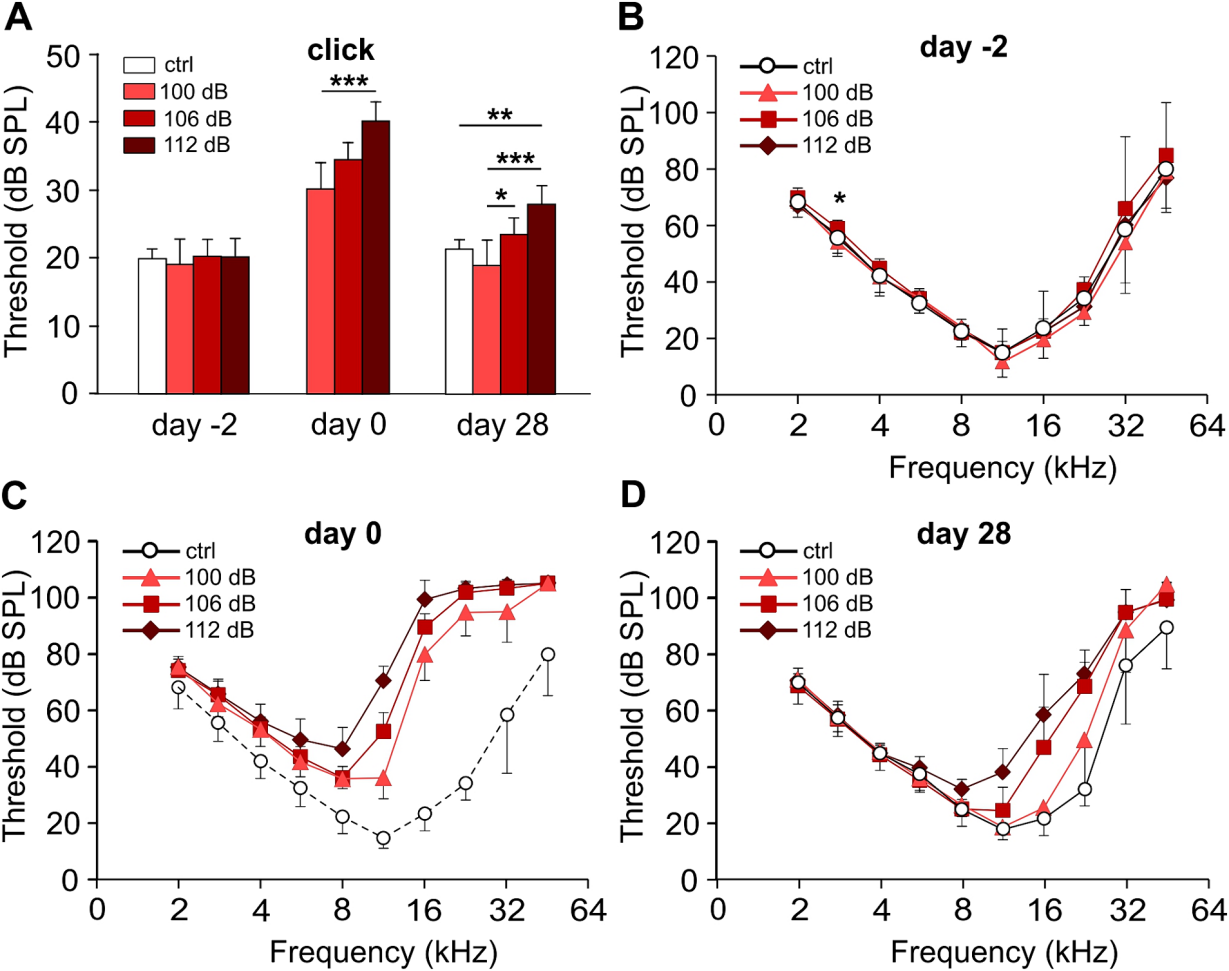
Hearing performance of the control and of the trauma groups before, directly after, and 28 days after noise trauma. Click ABR and pure-tone audiograms of the trauma groups before (day-2), directly after (day 0), and 28 days after the noise trauma (band noise, 8–16 kHz for 2 h) of either 100, 106, or 112 dB SPL and of the control group at days-2 and 28. **(A)** Click ABR thresholds (mean ± SD.) for the untreated control (ctrl) and the three trauma groups at days-2, 0, and 28. **(B–D)** Pure-tone audiograms showing mean f-ABR thresholds (±SD., only one direction is shown) recorded at day-2 **(B)**, after trauma (day 0, **C**), and at day 28 **(D)**. Data of the control group from day-2 are indicated in panel **(C)**, connected by dashed lines for comparison. The permanent threshold shift with respect to the untreated control 4 weeks after trauma was evaluated for each trauma strength and frequency **(D)**. Kruskal–Wallis test, with Bonferroni correction for multiple comparisons; **(A, B)** *p < 0.05; **p < 0.01; ***p < 0.001; statistical analysis for **(C,D)**, see Table 1 for clarity. Number of animals: day-2: control, 7; 100 dB, 9; 106 dB, 20; 112 dB, 10; day 0: 100 dB, 8; 106 dB, 20; 112 dB, 10; day 28: control, 7; 100 dB, 8; 106 dB, 20; 112 dB, 10.

We subjected C57BL/6 N mice to the classical noise trauma (band noise from 8 to 16 kHz for 2 h, 100 dB SPL). This trauma had been found to cause loss of synaptic ribbons and nerve fibers despite the lack of PTS in CBA/CaJ mice, which opened the field of noise-induced cochlear synaptopathy (Kujawa and Liberman, 2009). In addition, we also tested the effects of more intense noise (106 dB SPL and 112 dB SPL) on TTS and PTS, and on the fate of ribbons and postsynapses. In this study, we only show ABR data for days -2, 0, and 28. On day -2, click ABR thresholds were equal for the four experimental groups, including a control group that did not receive a trauma at day 0 except for one single frequency (2.8 kHz; Figure 2A). Click ABR thresholds increased monotonically with trauma strength at day 0, and declined 4 weeks after the trauma. Four weeks after trauma, click thresholds had returned to control values for the 100 dB SPL trauma but were still elevated for the 106 dB SPL and the 112 dB SPL trauma, respectively. Analysis of f-ABR thresholds before, directly after, and 4 weeks after the trauma (Figures 2B–D and Table 1) provides a frequency-specific view of TTS and PTS and the effects of trauma strength. f-ABR thresholds, which were nearly undistinguishable at day-2 (Figure 2B), strongly increased in the mid- to high-frequency region on the day of the trauma, indicating a TTS in the frequency band of the noise and above (Figure 2C and Table 1). For comparison, the threshold curve of the untreated control group is indicated by open symbols and a broken line. At day 28, the group exposed to 100 dB SPL had recovered with respect to the age-matched control group except for 45.2 kHz. The group exposed to 106 dB SPL, however, showed a PTS of 19 dB and 25 dB at 16 kHz and 22.6 kHz, respectively, whereas the 112 dB SPL trauma group exhibited a PTS in the entire range between 11.3 and 32 kHz, which was as large as 23 dB at 11.3 kHz and 36 dB at 16 kHz. For statistical analysis, see Table 1.

**Table 1:** Effect of trauma strength on f-ABR thresholds at day 0 (TTS) and day 28 (PTS).

Day 0											
Group	vs. group (dB SPL)	2 kHz	2.8 kHz	4 kHz	5.6 kHz	8 kHz	11.3 kHz	16 kHz	22.6 kHz	32 kHz	45.2 kHz
ctrl	100										**
	106		*	*	**	**	**	***	***	***	***
	112		*	**	***	***	***	***	***	***	**
100 dB SPL	106										
	112						***	**			
106 dB SPL	112					*	*				

Day 28											
Group	vs. group (dB SPL)	2 kHz	2.8 kHz	4 kHz	5.6 kHz	8 kHz	11.3 kHz	16 kHz	22.6 kHz	32 kHz	45.2 kHz
ctrl	100										*
	106							**	**		
	112						***	***	***		
100 dB SPL	106							*			
	112						***	**	*		
106 dB SPL	112					*					

Statistical analysis of inter-group comparisons of differences in f-ABR thresholds, see Figures 2C,D; Kruskal–Wallis test, with Bonferroni correction for multiple comparisons; *p < 0.05; ** *p* < 0.01; *** *p* < 0.001.

In summary, the 100 dB SPL trauma recapitulated a TTS trauma in our mouse background as described in Kujawa and Liberman (2009) for CBA/CaJ mice except for 45 kHz. Increasing the trauma strength to 106 and 112 dB SPL resulted in a stronger PTS with a wider range of affected frequencies as a monotonic function of trauma strength.

### Increased noise trauma leads to a stronger reduction of ABR wave I amplitudes

**Figure 3:**
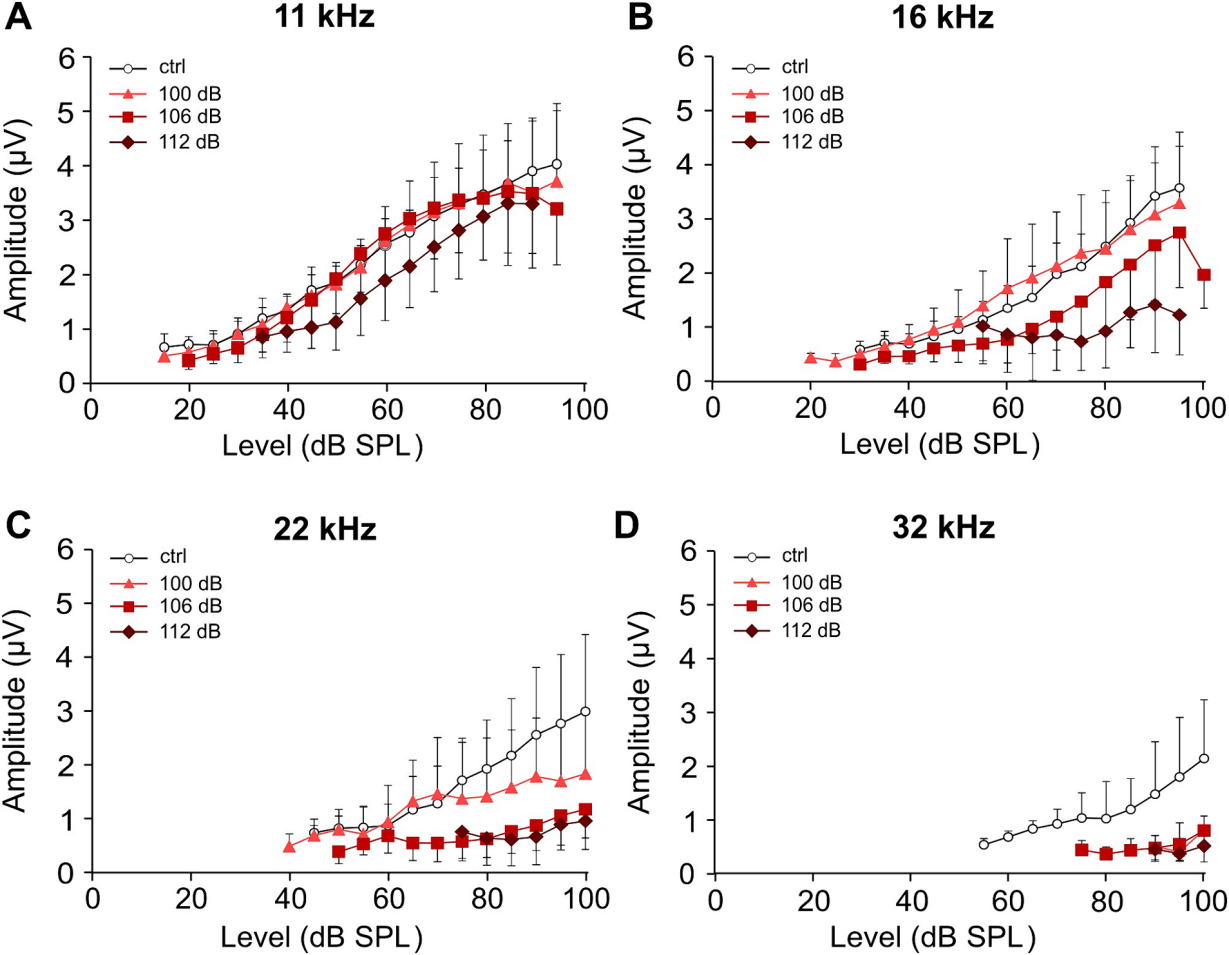
Effect of increasing strength of the noise trauma on growth functions of ABR wave I amplitudes 4 weeks after trauma. **(A–D)** Growth functions of the average peak-to-peak amplitudes of ABR wave I (±SD., only one direction is shown) for the control group (no trauma) and the groups exposed to 100 dB SPL, 106 dB SPL, and 112 dB SPL at 11 kHz **(A)**, 16 kHz **(B)**, 22 kHz **(C)**, and 32 kHz **(D)** at day 28. Note that because of the occurrence of permanent threshold increases, less amplitude values could be extracted for higher frequencies and higher trauma levels. The minimum number of data required for presenting a mean value was set to three in each condition.

Substantial failure of information transmission at IHC synapses due to the destruction of ribbons, peripheral dendrites, or both is not necessarily reflected by an increase in hearing thresholds (Kujawa and Liberman, 2009; Hickox et al., 2017). It is, however, reflected by the frequency-specific amplitude of ABR wave I, a readout for functional synapses between IHC ribbons and nerve fibers (Figure 3). Here, growth functions of averaged ABR peak-to-peak wave I amplitudes are shown for the frequencies 11.3, 16, 22.6, and 32 kHz in the untreated control group (Figure 3A) and for the three traumata (Figures 3B–D) at day 28, respectively. These growth functions indirectly also reflect the thresholds (starting point of a growth curve with respect to the level at the x-axis). Four weeks after trauma, the 100 dB SPL trauma led to (an irreversible) reduction of the growth functions at 22 kHz and 32 kHz only, whereas 106 dB SPL trauma additionally reduced the growth function at 16 kHz. Expectedly, the 112 dB SPL trauma showed the largest reduction of the growth functions for all four frequencies analyzed, including wave I amplitudes at 11 kHz.

### Increasing the noise trauma leads to the elevation of ABR wave I latencies

**Figure 4:**
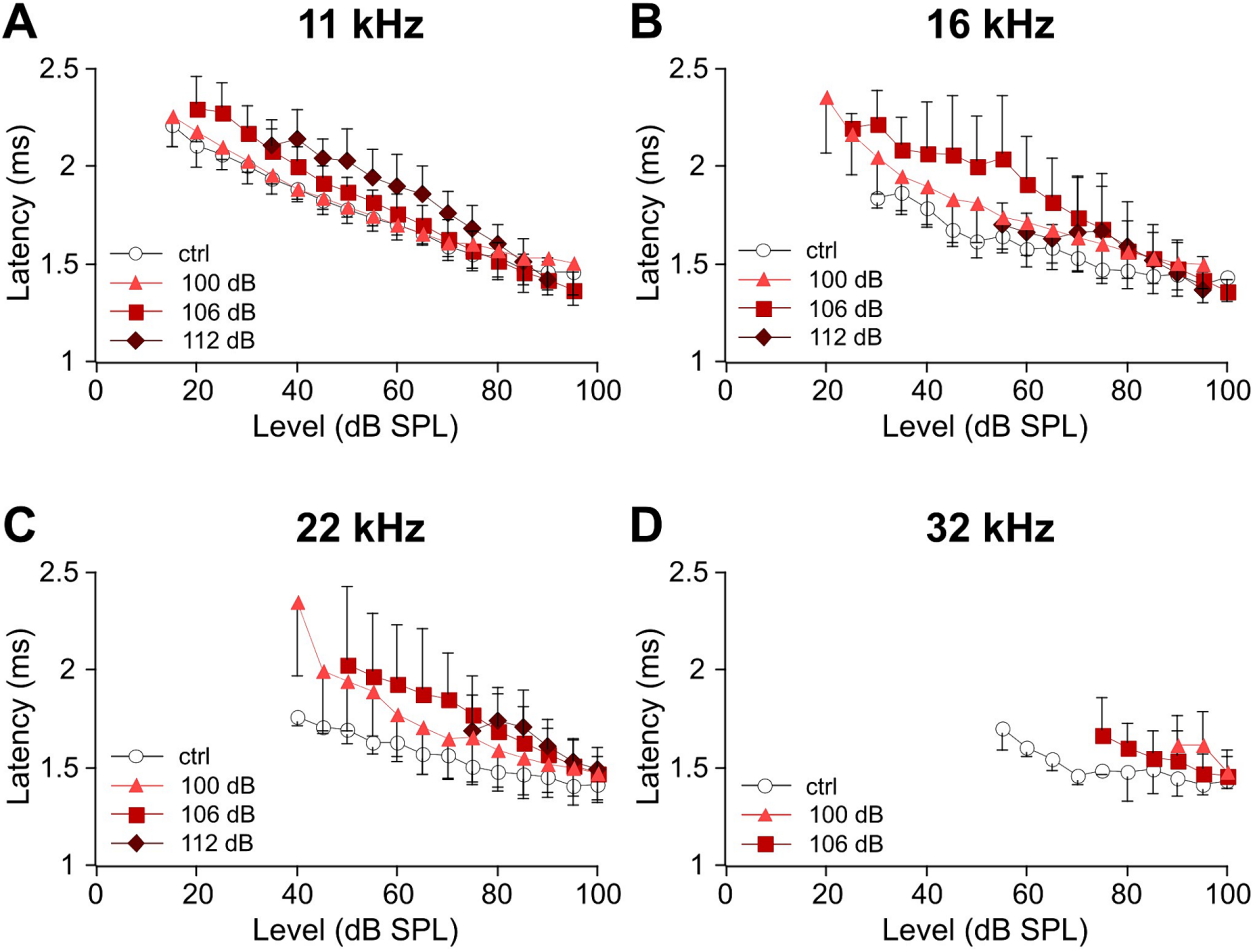
Sound level-dependent latencies of ABR wave I increase with trauma strength for the mid-to-high auditory frequencies. **(A–D)** Average level-dependent latencies of ABR wave I measured at the negative peak I (see Methods) are displayed for the unexposed control and the three trauma groups at day 28 after trauma for the frequencies 11 kHz **(A)**, 16 kHz **(B)**, 22 kHz **(C)**, and 32 kHz **(D)**. Note that because of the permanent threshold shift, fewer values were obtained for higher frequencies and higher trauma levels. The minimum number of data required for presenting a mean value was set to three in each condition.

The reduction of ABR wave I amplitudes (Figure 3) indicates a gradual loss of functional synaptic connections and afferent nerve fibers as a function of trauma strength in the mid- to high-frequency range. Next, we determined the latencies of wave I for the frequencies 11, 16, 22, and 32 kHz (Figure 4). It should be noted that under control conditions (i.e., without noise trauma), latencies increase with decreasing frequency as the traveling wave needs time to excite the basilar membrane at characteristic frequencies closer to the cochlear apex. Level-dependent ABR wave I latency functions were determined for the control and the three trauma groups (Figure 4). The shorter the latency at a fixed frequency and given level, the more HSR fibers are synchronously active (Bourien et al., 2014). Hence, noise trauma-induced loss of synaptic connections might increase latencies at a given level. At 11 kHz, there is a tendency for increased latencies after the 106 and the 112 dB trauma (Figure 4A). Latencies further deviate from the latency of the control group at 16 and 22 kHz (Figures 4B,C). Note a non-systematic level-dependent shape of the latency functions after the 112 dB SPL trauma at both 16 and 22 kHz, which will be addressed in the Discussion. At 32 kHz, latencies could hardly be measured at all (Figure 4D).

### Noise traumata did not cause outer hair cell loss

**Figure 5:**
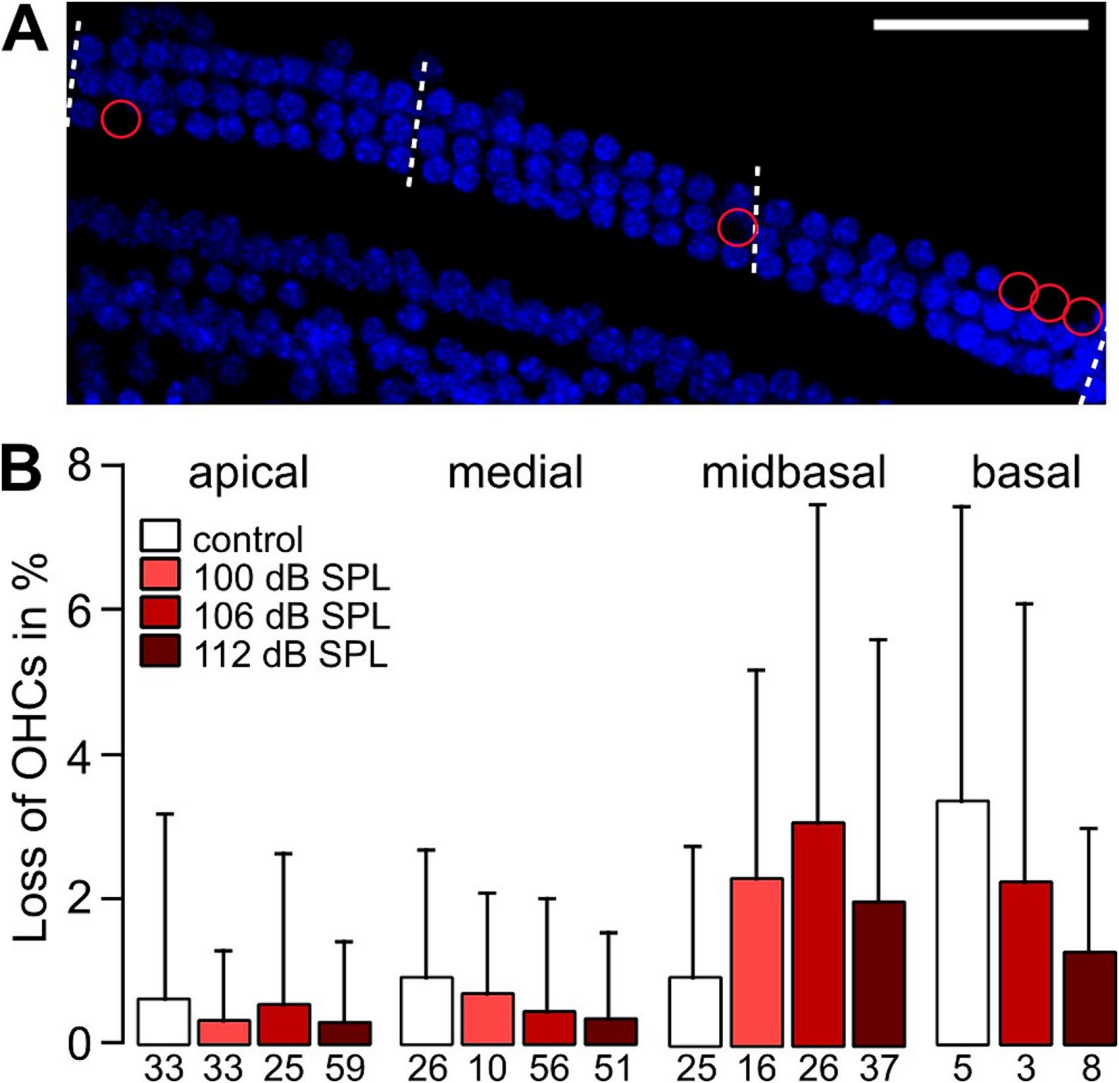
Average loss of OHCs in high-frequency cochlear regions is small and is not related to trauma strength. **(A)** Example MIPs of confocal stacks of the midbasal part of a whole-mount organ of Corti from a 12-week-old mouse that had been exposed to a 2 h noise trauma from 8 to 16 kHz of 100 dB SPL 4 weeks after trauma. The number of OHC nuclei (DAPI-stained, blue) was counted in regions of nominally 10 OHCs per row in length (i.e., nominally 30 OHCs for the three OHC rows) as indicated by the dashed lines. Missing nuclei are indicated by red circles. **(B)** Percentage of OHC loss in regions of 3 × 10 OHCs (mean + SD) for the control and the trauma groups as a function of cochlear location. Numbers below the bars indicate the number of regions of OHC counts providing the space for 30 OHCs obtained from 4 different mice. Scale bar: 50 μm.

We examined how outer hair cells (OHCs) and IHC synapses led to the malfunction of type I auditory nerve fibers (ANFs). OHCs amplify the vibrations of the basilar membrane and reduce the threshold for the mechanical activation of IHCs below ~50 dB SPL (Fettiplace, 2017). If part of the OHCs in a cochlear region are non-functional, the thresholds for activation of IHCs will be increased, which finally will also be reflected in altered ABR wave I growth functions. A previous study in CBA/CaJ mice showed massive OHC degeneration in the high-frequency region after a noise trauma of 100 dB SPL and an increase in distortion product otoacoustic emission (DPOAE) thresholds (Liberman et al., 2015). Although we did not analyze the function of OHCs by, e.g., measuring DPOAEs, we determined their presence by counting OHC nuclei in the apical, medial, midbasal, and basal region (*cf.* Figure 1B and see Methods) in DAPI-stained specimens that were also used for the analysis of IHC pre-and postsynapses. OHC numbers were assessed in regions of 10 slots (potential OHC place) × 3 rows. Figure 5A shows an example from a midbasal turn 4 weeks after the trauma of 100 dB SPL, with missing OHC nuclei encircled in red. Analyzing the percentage of OHC loss as a function of cochlear location and trauma strength (Figure 5B) revealed average numbers of <1% OHC loss in the apical and medial turn and of 1–3% with some variation in the midbasal and basal region, which did not depend on trauma strength, with the following *p*-values: 0.976 (apical), 0.142 (medial), 0.186 (midbasal), and 0.616 (basal part; Kruskal–Wallis test). Due to difficulties of preserving the delicate, narrow stretch of OHCs from the basal turn during dissection and labeling as well as the difficulties of imaging close to the bone there are no data for the basal turn in the 100 dB SPL trauma. In summary, there was no trauma-dependent excess loss of OHCs, even in the midbasal and basal cochlear regions. Although we cannot rule out that OHCs from noise trauma-exposed mice were less capable of active amplification 4 weeks after trauma, we conclude that the trauma-induced increase of hearing thresholds and the decrease of ABR wave I amplitudes were not caused by cellular OHC loss.

### Immunolabeling of ribbons and postsynapses

Classical noise-induced synaptopathy studies have focused on the detailed analysis of immunolabeled synaptic ribbons. Reduction of nerve fiber counts in CBA/CaJ mice pointed to a pronounced vulnerability of part of the ANF type I in the mid- to high-frequency regions to a 100 dB SPL trauma (Kujawa and Liberman, 2009). More recently, also postsynapses have been examined in rodents (Liberman et al., 2015; Kaur et al., 2019; Kim et al., 2019; Hickman et al., 2020; Manickam et al., 2023). Because of the variable performance of anti-GluR2/3 antibodies, we used Homer1 immunolabeling to characterize the number and size of postsynaptic receptor clusters in MIPs of cochlear whole-mounts. Homer1 is a scaffold of ionotropic glutamate receptors (Iasevoli et al., 2014) and is closely opposed to glutamate receptor clusters in afferents type I (Martinez-Monedero et al., 2016; Reijntjes et al., 2020). In the following experiments, we, therefore, used Homer1 as a proxy for the postsynapses and an indicator for a nerve fiber terminal close to the IHC basal pole.

**Figure 6:**
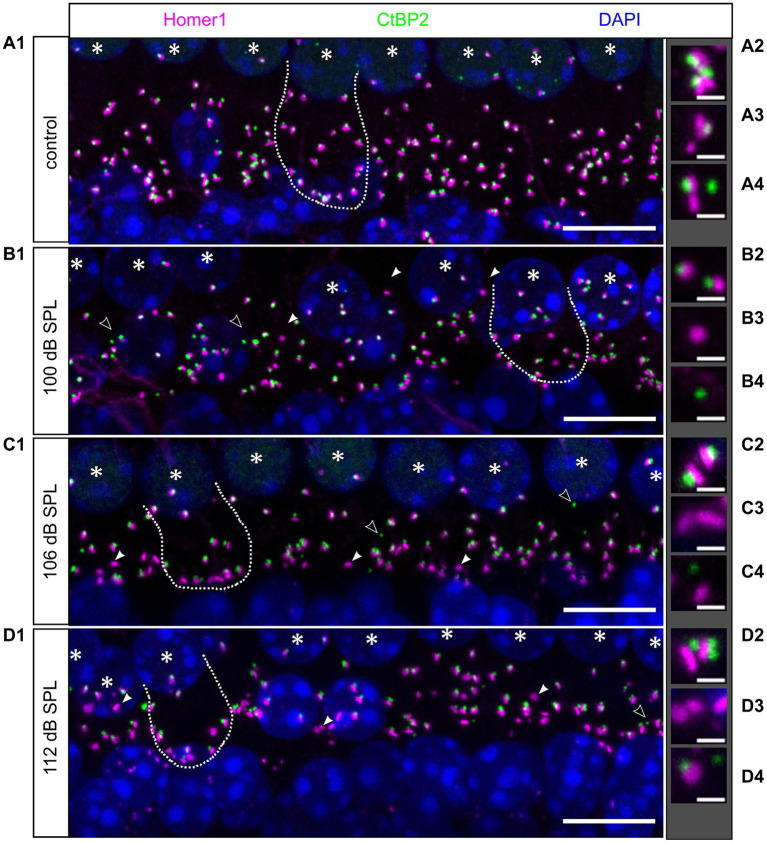
Effects of noise traumata of 100, 106, and 112 dB SPL on IHC synapses 4 weeks after trauma as evaluated by immunolabeling for CtBP2 and Homer1. MIPs of confocal stacks of whole-mount organs of Corti showing the basolateral pole of eight IHCs each immunolabeled for presynaptic ribbons (CtBP2, green) and postsynaptic glutamate receptors using Homer1 (magenta). **(A1)** Specimen of an unexposed 12-week-old control mouse. **(B1–D1)** Example specimen of mice that had been exposed to a noise trauma at 8 weeks of age of 100 dB SPL **(B1)**, 106 dB SPL **(C1)**, and 112 dB SPL **(D1)** at day 28 after trauma. Unpaired ribbons are indicated by open arrowheads and unpaired postsynaptic spots by closed arrowheads. Nuclei are stained in blue with DAPI; IHC nuclei are additionally indicated by white stars. An outline of one IHC is indicated by a white dotted line in each panel. The thumbnails to the right **(A2–A4,B2–B4,C2–C4,D2–D4)** show enlargements of paired synapses or unpaired synaptic components selected from the respective main panels **(A1–D1)**. Scale bars: 10 μm in main panels, 1 μm for thumbnails.

**Figure 7:**
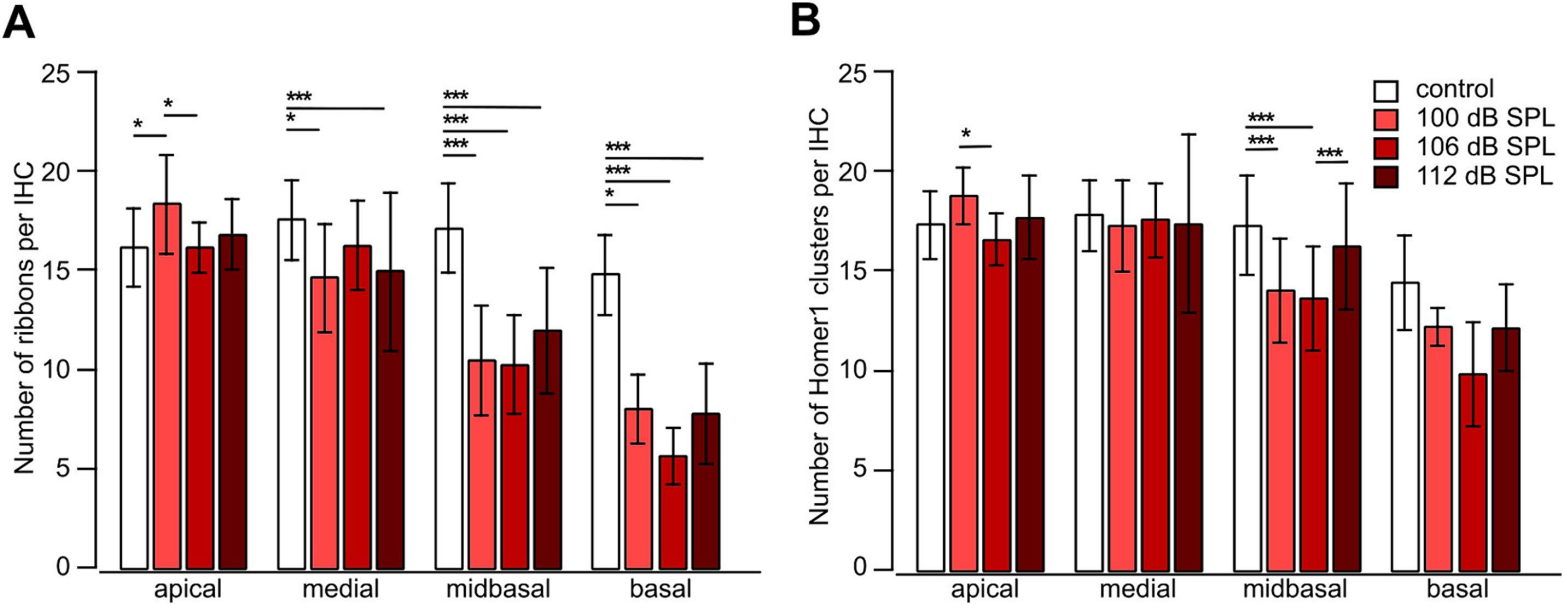
Noise trauma strongly reduces the number of presynaptic ribbons but less so the number of postsynaptic Homer1 clusters in the mid-to high-frequency range 4 weeks after trauma. **(A,B)** Number of presynaptic ribbons per IHC **(A)** and number of postsynaptic Homer1 clusters per IHC **(B)** as a function of trauma strength and cochlear location. Data are given as mean ± SD; numbers of regions comprising usually 8 IHCs: control (no trauma): apical 19, medial 37, midbasal 82, basal 12; 100 dB SPL: apical 8, medial 10, midbasal 15, basal 4; 106 dB SPL: apical 18, medial 33, midbasal 65, basal 4; 112 dB SPL: apical 46, medial 51, midbasal 48, basal 10, from 4 mice in total. One-way ANOVA for apical ribbons and medial postsynapses, Kruskal–Wallis test otherwise, with Bonferroni correction for multiple comparisons; *p < 0.05; **p < 0.01; ***p < 0.001.

Cochlear synaptopathy was characterized in mice euthanized 4 weeks after the respective trauma (day 28) and compared with aged-matched no trauma control mice. Imaging optical stacks of pieces of cochlear whole-mounts co-labeled for the ribbon marker CtBP2 and the postsynaptic marker Homer1 was performed in stretches comprising eight neighboring IHCs in all cochlear regions. Example MIPs of the midbasal region for no trauma control and for the different trauma strengths are shown in Figure 6. In general, pre-and postsynapses were closely apposed, often indicated by a white overlap of their fluorescence signals. Each trauma caused unpaired pre-and postsynapses, with a larger number of unpaired postsynapses. We quantified the number of pre-and postsynapses per IHC for the apical, medial, midbasal, and basal cochlear regions for the aged-matched no trauma control and the three traumata of 100 dB SPL, 106 dB SPL, and 112 dB SPL (Figure 7). A significant decrease of the average number of ribbons was observed in the medial region (for 100 dB and 112 dB) as well as in the midbasal and basal regions (for all three traumata, respectively; Figure 7A). Four weeks after the 100 dB SPL trauma, ribbon number declined from 17.5 ± 2.0 to 14.6 ± 2.7, corresponding to 83.5% in the medial region, from 17.1 ± 2.3 to 10.4 ± 2.8 or 61.0%, in the midbasal region, and from 14.8 ± 2.0 to 8.0 ± 1.7 or 53.9%, in the basal region. Notably, increasing the trauma to 106 dB SPL or 112 dB SPL did not aggravate the ribbon loss, suggesting that all vulnerable ribbons were fully destroyed by the 100 dB SPL trauma. Regarding the postsynapses, a significant loss was only observed in the midbasal region for the 100 dB SPL trauma (from 17.3 ± 2.5 to 14.0 ± 2.6 or 81.1%) and the 106 dB SPL trauma (to 13.6 ± 2.6 or 80.0%; Figure 7B). Surprisingly, the 112 dB SPL trauma did not cause a significant decline of Homer1 clusters. Similarly, in the basal cochlear region, there was no significant decline in the number of postsynaptic clusters for any of the traumata.

Taken together, presynapses were more vulnerable than postsynapses to an acoustic trauma ranging from 100 dB SPL to 112 dB SPL in the mid- to high-frequency regions of the cochlea.

**Figure 8:**
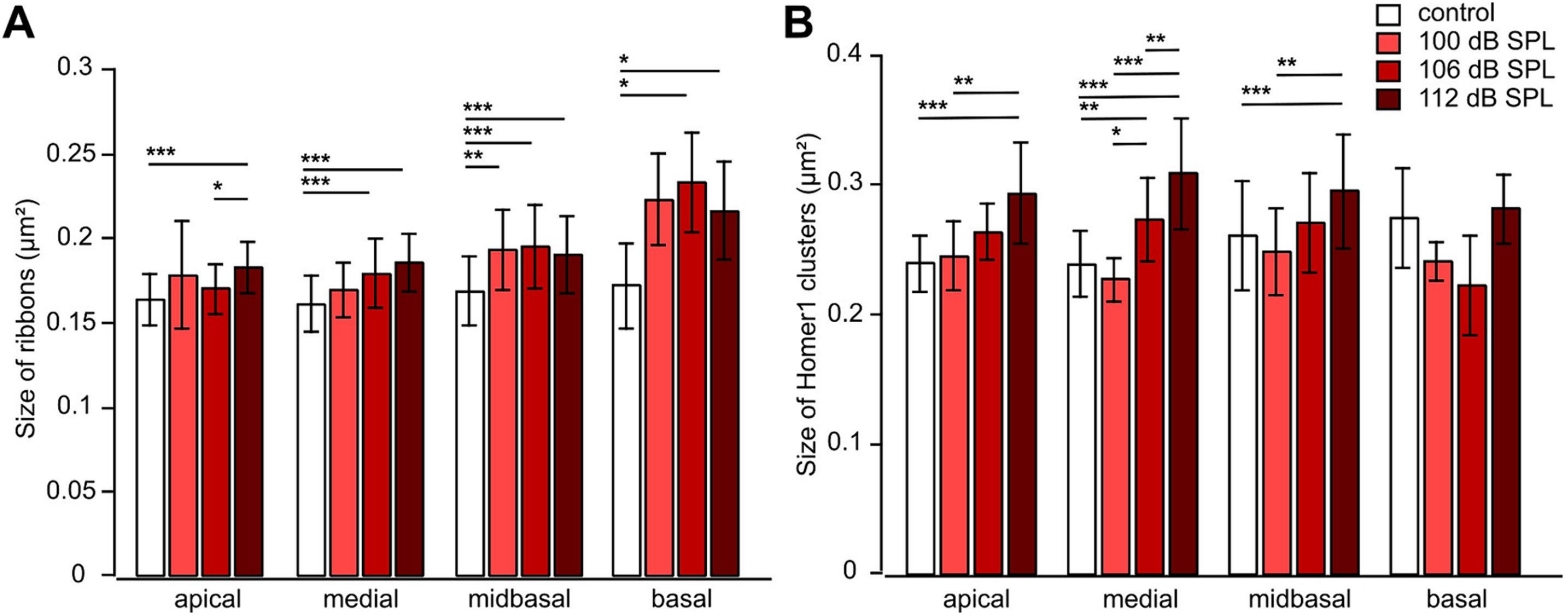
Noise trauma increases the sizes of both presynaptic ribbons and Homer1 clusters 4 weeks after trauma. **(A,B)** Mean area ± SD of presynaptic ribbons **(A)** and mean area ± SD of postsynaptic Homer1 clusters **(B)** Figure 6 as a function of trauma strength and cochlear location. Data are given as mean ± SD; numbers of regions comprising usually 8 IHCs: control (no trauma): apical 19, medial 37, midbasal 82, basal 12; 100 dB SPL: apical 8, medial 10, midbasal 15, basal 4; 106 dB SPL: apical 18, medial 33, midbasal 65, basal 4; 112 dB SPL: apical 46, medial 51, midbasal 48, basal 10, from 4 mice in total. Kruskal–Wallis test with Bonferroni correction for multiple comparisons, * *p* < 0.05; ** *p* < 0.01; *** *p* < 0.001.

We also analyzed the effects of acoustic trauma on the sizes of pre-and postsynapses as judged from their area in MIPs (Figure 8). The average area of ribbons significantly increased with trauma even in the apical region (for 112 dB SPL), medial region (for both, 106 and 112 dB SPL), midbasal region (for all trauma strengths), and basal region (for both 106 and 112 dB SPL; Figure 8A). Unlike the loss of ribbons, the increase in the size appeared to scale with trauma strength. We also observed an increase in the postsynaptic area except for the basal region (Figure 8B). The increase in the size of Homer1 clusters was not as large as in the case of ribbons but appeared to scale with trauma strength, as it was very prominent and highly significant for the highest trauma applied (112 dB SPL) in the apical, medial, and midbasal regions (Figure 8B).

**Figure 9:**
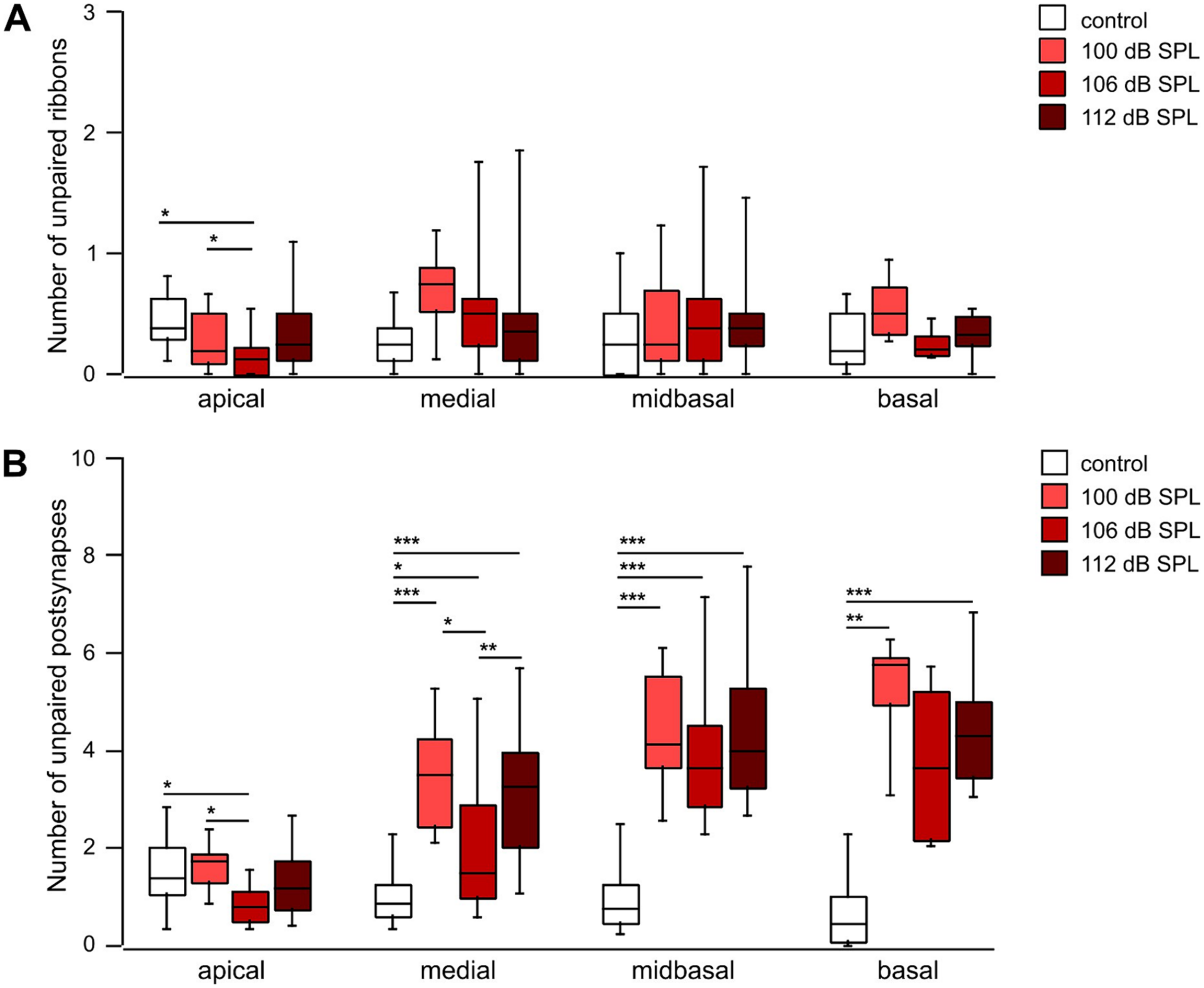
Postsynapses are less vulnerable to noise trauma than presynaptic ribbons **(A,B)** Box-and-whisker plots of the number of unpaired ribbons per IHC **(A)** and of orphan postsynapses per IHC (Homer1 clusters, **B**) as a function of cochlear location 4 weeks after trauma of the respective strength. Regions with usually 8 IHCs that went into the analysis of Figures 7, 8 were further analyzed for the number of unpaired pre-and postsynapses. Age-matched groups that did not receive any trauma, but were tested for their hearing function at days -2 and 28, served as controls. One-way ANOVA for unpaired ribbons of the midbasal and basal region; Kruskal–Wallis test otherwise, Bonferroni correction for multiple comparisons, * *p* < 0.05; ** *p* < 0.01; *** *p* < 0.001.

Finally, we determined the number of unpaired ribbons/IHC and of unpaired postsynapses/IHC as a function of trauma strength and cochlear location (Figure 9). Though the number of unpaired ribbons/IHC was quite variable after trauma, the median number ranged between 0.13 and 0.75 for control or any trauma condition along the cochlear axis (Figure 9A). Unpaired ribbons thus were a rare finding for all conditions (no trauma control, traumata of 100 dB SPL, 106 dB SPL, and 112 dB SPL) in the entire cochlea (Figure 9A). In contrast, trauma increased the number of unpaired (‘orphan’) postsynapses in the medial, midbasal, and basal regions irrespective of trauma strength. Whereas in control mice the median number of unpaired postsynapses ranged between 0.75 and 1.38, the 100 dB SPL trauma caused 1.75 (apical region) to 5.75 orphan postsynapses per IHC (basal region, Figure 9B). Notably, increasing the trauma strength to 106 or 112 dB SPL did not further increase the number of orphan postsynapses. It should be noted that the loss of ribbons was generally larger than the number of orphan postsynapses. The 100 dB SPL trauma, e.g., caused loss of 6.7 ribbons per IHC (Figure 7A) and generated 4.2 orphan postsynapses per IHC (Figure 9B), indicating that on average only 2-3 postsynapses (i.e., nerve terminals) had degenerated per IHC.

Taken together, the mildest trauma applied (100 dB SPL) caused a large reduction of the number of ribbons in the medial (by 26%), midbasal (39%) and basal cochlear region (46%) and a small reduction of the number of postsynapses in the midbasal cochlear region (by 19%), both of which were not aggravated by a further increase in trauma strength to 106 dB SPL or even to 112 dB SPL. As a consequence, a substantial number of postsynapses in the mid- to high-frequency region was left without a presynaptic partner in each trauma (orphan postsynapses). In contrast, unpaired ribbons were rare—the median value was well below one per IHC in both control and trauma conditions, underlining the specific noise-induced vulnerability of IHC synaptic ribbons.

## Discussion

Using C57BL/6 N mice, we have shown that a 100 dB SPL, 8–16 kHz, 2 h noise trauma causes a TTS ranging from 8 to 45 kHz and a PTS restricted to 45 kHz compared with age-matched control animals (without trauma) at day 28. Increasing the trauma level to 106 and 112 dB SPL aggravated the degree of both TTS and PTS and enlarged the permanently affected frequency range toward lower frequencies, respectively. All traumata reduced the amplitudes of ABR wave I in the 16 kHz–32 kHz region. On the ultrastructural level, a loss of the number of synaptic ribbons in the medial, midbasal, and basal regions was observed after the 100 dB SPL trauma that did not further increase with trauma strength. The reduction of postsynaptic Homer1 clusters was less severe for all traumata. As a consequence, 4–6 unpaired (orphan) postsynapses per IHC were present in the mid- to high-frequency region, a phenomenon that did not scale with trauma strength.

### Noise-induced changes in hearing thresholds

Here, we used a mouse line (C57BL/6 N) that shows early onset of high-frequency hearing loss starting at the age of 3–6 months, which is caused by a cadherin 23 splice variant that has a slight but progressive impact on tip link stability (Zhou et al., 2006; Kane et al., 2012; Jeng et al., 2020b). Within the time course of our experiments, from 2 to 3 months of age, this effect was negligible in the control group (without trauma). Four weeks after trauma, the click thresholds were unaffected for both the 100 dB SPL and the 106 dB SPL trauma, respectively, yet significantly increased after the 112 dB SPL trauma (Figure 2A). HHL per definition describes the loss of functional IHC synapses/nerve fibers without a permanent threshold elevation. Across ABR test frequencies, we observed a threshold elevation at 45 kHz 4 weeks after the 100 dB SPL trauma compared with unexposed age-matched control animals, indicating that in the highest frequency range the hearing loss was overt rather than hidden. Depending on trauma strength, duration, and frequency characteristics, various cochlear structures can be permanently affected. It was until the discovery of hidden hearing loss that OHCs were regarded as the most sensitive structures in the cochlea (Wang et al., 2002; Liberman and Kujawa, 2017; Natarajan et al., 2023). In CBA/CaJ mice, massive OHC loss was observed after the classical 100 dB SPL trauma (8–16 kHz, 2 h) in the basal region (Liberman et al., 2015). In this study, trauma strength did not correlate with the cellular loss of OHCs 4 weeks after the trauma (Figure 5) but the possibility of a trauma-related *functional* OHC impairment, which would feed less energy into the cochlea, should be considered. A noise-induced functional impairment of OHCs may have been responsible for our high-frequency PTS of the 100 dB SPL trauma, possibly caused by the cadherin 23 malfunction (Zhou et al., 2006; Kane et al., 2012; Jeng et al., 2020b) or other OHC susceptibility genes (Lavinsky et al., 2021a; Shuster et al., 2021).

The degree of both TTS and PTS and the range of affected frequencies were monotonic functions of trauma strengths (Figures 2C,D and Table 1), similar to findings in CBA/CaJ mice exposed to the same trauma paradigm with either 100, 106, 112, or 116 dB SPL (Wang et al., 2002). Four weeks after the 112 dB SPL trauma, a small but significant PTS remained down to 11.3 kHz, the center of the noise frequency band (Figure 2D). Using a less intense noise trauma, the PTS is usually shifted by half an octave toward high-frequency (basal) regions because of cochlear mechanics (Cody and Johnstone, 1981). In our 112 dB SPL trauma experiments, basilar membrane movements were so large that the PTS extended down to 11.3 kHz.

### Noise-induced changes of ABR wave I amplitudes and latencies

Growth functions of ABR wave I amplitudes reflect level-dependent synchronous firing of ANFs. With increasing level of the stimulus, the fiber types Ia, Ib, and Ic should be activated in this sequence, and reduced growth functions indicate a loss of functional nerve fibers (Moser et al., 2023). In this study, ABR growth functions were reduced for frequencies ≥22 kHz (for the 100 dB SPL trauma), ≥ 16 kHz (for the 106 dB SPL trauma), and ≥11 kHz (for the 112 dB SPL trauma) 4 weeks after trauma (Figure 3) indicating an increasing loss of functional fibers with trauma strength.

We further show latency functions of wave I for four selected frequencies, which indicate that at a given frequency latencies mostly rose with increasing trauma strength (Figure 4). In a healthy cochlea, both amplitude and latency of compound action potentials of the auditory nerve, the basis of the ABR signal, are dominated by HSR fibers (Bourien et al., 2014). Cochleae of different mouse lines treated with a similar HHL trauma showed a reduction in synaptic pairs by ~50% (Kujawa and Liberman, 2009; Suthakar and Liberman, 2021). These numbers are too high for exclusive functional type Ic fiber loss. Indeed, noise-induced loss of type Ia fibers has been recently demonstrated in CBA/CaJ mice (Suthakar and Liberman, 2021).

### Factors affecting the consequences of noise trauma

Recently, genetic studies on hearing performance and the vulnerability to noise trauma have been performed for a large number of mouse inbred strains (Boussaty et al., 2020; Jeng et al., 2020a, 2020b; Lavinsky et al., 2021b; Milon et al., 2021; Early et al., 2022). In genetic noise studies, neither CBA/CaJ nor C57BL/6 N mice were included, only the line C57BLKS/J, which is closely related to the C57BL/6 J strain (Lavinsky et al., 2021b). It should be kept in mind that the C57BL/6 N line was separated from C57BL/6 J mice in 1951 (Kane et al., 2012). With regard to the stability of wave I amplitude, C57BLKS/J mice were found to be rather resistant to noise trauma from 2 to 10 kHz at 108 dB SPL (Lavinsky et al., 2021b). The C57BL/6 N line is the basis for genetic mouse models generated by ‘The International Knockout Mouse Consortium’ (Mianné et al., 2016) and is therefore important for ourselves and others (Jeng et al., 2020a, 2020b).

Apart from genetic differences, a second factor that may affect the outcome of noise trauma experiments is anesthesia, which may alter the middle ear reflex or cochlear efferent reflexes (Ohlemiller et al., 2016). There are few studies in which the effects of noise trauma in awake mice were compared with those of anesthetized mice (Reijntjes et al., 2018; Jongkamonwiwat et al., 2020). One study did not find a difference in TTS or PTS in f-ABR as well as in the number of ribbons/IHC in all cochlear regions for C57BL/6 J and FVB mice (Reijntjes et al., 2018). Another study observed a larger TTS in anesthetized FVB mice after 7–14 days, which came down to the same PTS thresholds as in awake mice for 94, 100, and 105 dB SPL traumata, respectively (Jongkamonwiwat et al., 2020). Both groups used ketamine-based anesthesia, whereas we used fully antagonizable fentanyl-based anesthesia to limit its duration in frequent ABR measurements.

### Cochlear synaptopathy—IHC ribbons are more vulnerable than AN fibers

A noise trauma that causes overt rather than hidden hearing loss (i.e., PTS) includes cochlear synaptopathy (Wu et al., 2020, 2021), but so far, little systematic quantification as a function of trauma strength has been performed. Noise-induced cochlear synaptopathy is defined as the loss of paired IHC synapses at a certain time after a noise trauma, leading to the reduction of auditory information channels between IHCs and the central auditory system. At the level of a single IHC-SGN synapse, it can present as (i) selective loss of the ribbon, (ii) selective loss of the postsynapse, (iii) loss of both ribbon and postsynapse, or (iv) detachment of ribbon and postsynapse without degeneration. In all four constellations, the transmission of information along the specific AN fiber will be prevented. The accepted hypothesis is that acoustic overstimulation of IHCs causes glutamate excitotoxicity in the postsynapses of type I (predominantly Ic, i.e., LSR) fibers causing swelling, bursting, and retraction of the terminal dendrite from the IHC (Ruel et al., 2007; Liberman and Kujawa, 2017; Valero et al., 2017; Kim et al., 2019; Hu et al., 2020). On the other hand, synaptic ribbons are vulnerable to acoustic overstimulation, too—with loss of up to 50–60% on the day after exposure to 100 dB SPL in the 32 kHz region in CBA/CaJ mice (Kujawa and Liberman, 2009; Liberman et al., 2015). Studies using mouse lines such as C57BL/6 J showed some degree of ribbon regeneration (Shi et al., 2015; Kaur et al., 2019; Kim et al., 2019), whereas in guinea pigs, most ribbons regenerated after noise trauma (Shi et al., 2013, 2016; Hickman et al., 2020, 2021).

We used the postsynaptic marker Homer1, which is a scaffold for glutamate receptors (Iasevoli et al., 2014) because anti-Homer1 antibody labeling, which indicates the existence and position of the afferent bouton, is very robust (Martinez-Monedero et al., 2016; Reijntjes et al., 2020). An unexpected finding of this study is that more ribbons than postsynapses were lost 4 weeks after trauma leading to 3–6 unpaired postsynapses per IHC in the medial, midbasal, and basal regions. This reveals that despite the presumed excitotoxic swelling of afferent boutons, more boutons than ribbons survived 4 weeks after trauma, indicating that IHC synaptic ribbons rather than AN fibers are the most vulnerable structures in C57BL/6 N mice. We cannot tell whether the trauma-induced orphan terminals would have retracted and degenerated over many months after their partner ribbon had died, as observed in CBA/CaJ mice (Kujawa and Liberman, 2009) because the analysis of the long-term survival of SGN was not the aim of our study. Moreover, C57BL/6 N mice are not suitable for following hearing into old age. Nevertheless, it will be interesting to look at the fate of synapses at time points closer to the trauma (e.g., on days 1, 3, 7, and 14), which we plan in the future.

The striking vulnerability of part of synaptic ribbons poses new questions as to (i) which ribbons are primarily affected and (ii) what is the mechanism of degeneration. Answering these questions may foster therapies for treating noise-induced cochlear synaptopathy in humans by addressing a novel target, the *presynaptic ribbons*. So far, treatment aimed at the regeneration of ANF by intracochlear application of neurotrophin 3 in a mouse model (Suzuki et al., 2016; Ji et al., 2024).

The finding that surviving ribbons on average had a larger size (Figure 7) may suggest either compensation for the substantial loss in ribbon number (hence in functional connections to the brain) by increased ribbon sizes or a process of dysregulation. An increase in ribbon size has been found in previous studies (Liberman et al., 2015; Kim et al., 2019; Reijntjes et al., 2020), which suggests some presynaptic plasticity on the IHC side. This leads to the question as to why IHCs are able to change the size of surviving or newly built ribbons rather than replacing dead ribbons with new ones at those sites that oppose orphan boutons. Electron microscopic studies revealed a structural phenotype in presynaptic regions of noise-damaged IHCs, specifically a dysregulation of the vesicle recycling pathway (Bullen et al., 2019; Moverman et al., 2023). The range of dysregulation may span from nearly normal functioning ribbons with slightly changed sizes to failure of membrane recycling, preventing the build-up of new ribbons replacing damaged ones.

Noise-induced changes pointing to plasticity or dysregulation are also present in postsynapses, as shown by a trauma-induced increase in size (Figure 8B; Bullen et al., 2019) and upregulation of PSD-95 (Bao et al., 2004). A recent analysis of noise-induced transcriptomic changes in whole cochlea and specific cell types such as OHCs and SGN type Ia found activation of the immune response and many cell-type specific changes, especially the ATF3/ATF4 stress-response pathway (Milon et al., 2021).

Another unexpected finding was the fact that the loss of ribbons in the most affected (midbasal) region 4 weeks after the 100 dB SPL trauma could not be aggravated by further increasing trauma strength, as was the number of orphan postsynapses (for the medial, midbasal, and basal regions). In other words, the 100 dB SPL trauma that did not change ABR thresholds (except the threshold at 45 kHz in this study) was large enough to damage all the vulnerable ribbons such that higher traumata did not add further damage to the synapses.

In humans, evidence is accumulating that an increase in sensorineural hearing thresholds might almost always be accompanied by a considerable degree of cochlear synaptopathy. In the aging process, loss of information channels from IHCs to the central nervous system is likely a major cause for poor speech recognition abilities, especially in background noise, and is exacerbated by the individual noise history (Bakay et al., 2018; Wu et al., 2019, 2021). Accepting that the IHC synapse is the most vulnerable structure in the cochlea endangered by both noise and aging, which leads to severe communication problems on top of increasing hearing thresholds, requires more effort in both protection measures and in developing therapeutic strategies targeting IHC synapses, especially presynaptic ribbons.

## Data Availability

The raw data supporting the conclusions of this article will be made available by the authors, without undue reservation.
